# Evaluating Neural Network Performance in Predicting Disease Status and Tissue Source of JC Polyomavirus from Patient Isolates Based on the Hypervariable Region of the Viral Genome

**DOI:** 10.3390/v17010012

**Published:** 2024-12-25

**Authors:** Aiden M. C. Pike, Saeed Amal, Melissa S. Maginnis, Michael P. Wilczek

**Affiliations:** 1 Maine Space Grant Consortium, Augusta, ME 04330, USA; pike.aid@northeastern.edu; 2 Life Sciences, Health, and Engineering Department, The Roux Institute, Northeastern University, Portland, ME 04101, USA; 3 Department of Molecular and Biomedical Sciences, University of Maine, Orono, ME 04469, USA; melissa.maginnis@maine.edu; 4 The Roux Institute, Northeastern University, Portland, ME 04101, USA; s.amal@northeastern.edu; 5 Department of Bioengineering, College of Engineering, Northeastern University, Boston, MA 02115, USA; 6 Graduate School in Biomedical Science and Engineering, University of Maine, Orono, ME 04469, USA; 7 Observational Health Data Sciences and Informatics Center, The Roux Institute, Northeastern University, Portland, ME 04101, USA; 8 Department of Chemistry and Chemical Biology, College of Science, Northeastern University, Boston, MA 02115, USA

**Keywords:** JC polyomavirus, non-coding control region, *k*-mer, machine learning, neural network, multilayer perceptron, classification, singular value decomposition

## Abstract

JC polyomavirus (JCPyV) establishes a persistent, asymptomatic kidney infection in most of the population. However, JCPyV can reactivate in immunocompromised individuals and cause progressive multifocal leukoencephalopathy (PML), a fatal demyelinating disease with no approved treatment. Mutations in the hypervariable non-coding control region (NCCR) of the JCPyV genome have been linked to disease outcomes and neuropathogenesis, yet few metanalyses document these associations. Many online sequence entries, including those on NCBI databases, lack sufficient sample information, limiting large-scale analyses of NCCR sequences. Machine learning techniques, however, can augment available data for analysis. This study employs a previously compiled dataset of 989 JCPyV NCCR sequences from GenBank with associated patient PML status and viral tissue source to train multilayer perceptrons for predicting missing information within the dataset. The PML status and tissue source models were 100% and 87.8% accurate, respectively. Within the dataset, 348 samples had an unconfirmed PML status, where 259 were predicted as No PML and 89 as PML sequences. Of the 63 sequences with unconfirmed tissue sources, eight samples were predicted as urine, 13 as blood, and 42 as cerebrospinal fluid. These models can improve viral sequence identification and provide insights into viral mutations and pathogenesis.

## 1. Introduction

JC polyomavirus (JCPyV) is a ubiquitous human pathogen that infects most of the adult population with a greater seroprevalence among men [1,2,3] by establishing a persistent infection and can cause a fatal, incurable disease [4,5]. In immunocompetent patients, JCPyV establishes an asymptomatic infection of the kidneys and can be detected in the urine [6,7,8,9,10]; however, in immunosuppressed individuals, the virus can reactivate and spread within the central nervous system (CNS) to cause progressive multifocal leukoencephalopathy (PML), a fatal demyelinating disease characterized by the lysis of astrocytes and oligodendrocytes [11,12,13,14]. PML has been found to occur in patients with HIV, those taking immunomodulatory therapies for immune-mediated diseases such as multiple sclerosis, organ transplant recipients, and those with hematological diseases [15,16,17,18]. There are no currently approved therapies for JCPyV infection or PML, including a prophylactic vaccine [19], requiring treatment methods to address the underlying immunocompromised status of the patient to restore the immune system [15]. However, understanding the genomic signatures of JCPyV in relation to disease outcomes could provide additional information for disease prognosis or clinical management.

JCPyV is a nonenveloped virus with a circular double-stranded DNA genome that is ~5100 base pairs (bp) with divergently encoded arms of early and late genes separated by the non-coding control region (NCCR) [14]. The early genes are responsible for establishing an environment conducive to viral replication in the host cell and consist of the large tumor antigen (TAg) with TAg splice variants and the small tumor antigen (tAg) [10]. The late genes encode the agnoprotein, followed by the structural proteins viral protein (VP) 1, VP2, and VP3 [10]. Genetic variations throughout the JCPyV genome, such as within the structural proteins or intergenic region between VP1 and TAg, can be used to classify the virus into seven major geographic genotypes (Types 1 through 8, excluding 5) that correspond with distinct human populations [20]. Generally, genotypes 1 and 4 are associated with European populations, types 2 and 7 with Asian and Amerindian populations, types 3 and 6 with African populations, and type 8 with Oceanic populations [21,22,23]. The NCCR is roughly 400 bp and contains highly conserved genomic features, including the origin of replication and transcriptional elements of early genes [6,24,25]. The NCCR, situated between the early and late genes, is roughly 400 bp and is defined as the region between the start codons of the T antigen and agnoprotein, which harbors sequences for regulating viral replication [6,24].

As with other DNA viruses, JCPyV relies on the host transcriptional machinery for replication. The early proximal portion of the NCCR is highly conserved as it contains the origin of replication and elements for the transcription of early genes [6,25]. The late proximal region of the NCCR is a highly variable region that can undergo frequent recombination and consists of six unique blocks labeled “a” through “f” [14]. The archetypal, or non-pathogenic, strain contains all six blocks in alphabetical order and is primarily found in the kidneys and urine of healthy patients but can also be isolated from PML patients [9,12,26]. The archetypal strain is the predominant strain isolated from urine, though other rearranged NCCR sequences have been found [27,28,29]. Viral isolates in PML patients from sources other than urine, such as the blood or cerebrospinal fluid (CSF), often contain highly mutated NCCR sequences and exist in a quasispecies population [29,30]. The prototypal JCPyV isolate Mad-1 was the first to be isolated from a PML patient and is composed of tandem repeats of blocks “a”, “c”, and “e” with a final “f” block [12,30,31]. Rearrangements of the NCCR frequently occur in patients with PML, which can increase or decrease the occurrence of transcription factor binding sites that orchestrate viral transcription and subsequent replication [20,30,32,33]. Moreover, NCCR rearrangements have been suggested to expand JCPyV cellular tropism, promote neuropathogenesis, and increase viral replication [28,34]; however, it remains unclear whether NCCR rearrangements are required for or are a result of neuroinvasion [35].

Although a relationship between NCCR mutations and PML has been established and documented [29,30], few comprehensive analyses relate NCCR sequences to clinical manifestations and tissue location. A meta-analysis from the Maginnis laboratory previously compiled 989 published NCCR nucleotide sequences isolated from deidentified patient samples from the NCBI Nucleotide database to explore how host transcription factor binding sites in the NCCR are influenced through rearrangements [32]. The NCCR sequences were defined by the tissue from which the sequence was isolated and the PML status of the patient [32], yet many of the samples either lack or report imprecise labels, which complicates further analysis into the connection between NCCR mutations and the patient’s PML status or viral tissue source.

To address the challenges associated with classifying large volumes of biological data, many have employed machine learning (ML) techniques to process information and apply relevant predictions, such as using a neural network (NN) [36,37,38,39,40,41,42,43,44,45,46,47,48]. Previous research has shown that NNs are more capable of learning expressive and accurate data representations than traditional machine learning methods [49]. Given the prevalence of image-based data in the biomedical field, many studies integrating NN capabilities focus on the implementation of convolutional neural network (CNN) architectures for data classification due to computational efficiency, scalability, and ability to analyze data with spatial dependencies [50], either by using raw images or developing image representations of biological data [40,41,42,43,44,45,46]. However, there exist other NN architectures capable of accurate classification, such as a multilayer perceptron (MLP), which is a class of flexible networks capable of learning many different types of data [51] and does not inherently rely upon local spatial relationships within the data.

When using an NN to analyze nucleotide sequence data, there is a challenge between nucleotide sequences and NN functionality. Neural networks require numerical data of uniform dimensions as input, while nucleotide sequences consist of a variable-length string of letters [51,52,53]. To overcome the barrier between biological data and NN learning, the features of the nucleotide sequence are mapped into a numerical space using an encoding method to make the information comprehensible to machines. One possible option for encoding genomic data is to one-hot encode nucleotides. Although this process directly converts nucleotide sequences into NN-comprehensible information, the sequence length remains variable [54]. Another common technique is to convert the nucleotide sequence into *k*-mer representations, which can be used to construct highly dimensional numerical data [36,39,41,43,44]. This can include creating image representations [42], *k*-mer tokenization [36], and semantically embedded vectors [55], which produce uniform length but do not retain positional information. Though the information on the nucleotide sequence can then be transmitted through the NN, it remains obfuscated in terms of visualization due to the highly dimensional nature of the data. Using a tool such as singular value decomposition (SVD), the dimensionality of the data can be reduced while retaining key variance, facilitating the visualization of the data to elucidate the underlying features [56].

Machine learning also comes with the obstacle of ensuring the model can adequately learn the relationships within the training data and generalize to new data. Machine learning models, including NNs, can suffer from overfitting, where the model learns the noise in the training data, resulting in highly accurate predictions of training data but an inability to generalize to new data [57]. Causes of model overfitting can include using a small training dataset [58], imbalanced classes in the training data [59], and model complexity [60]. There are several strategies to prevent models from overfitting, including early stopping, node dropout, and dataset balancing [61,62,63]. Early stopping can prevent models from overfitting to training by using a stop criterion to cease model fitting, such as a growing disparity in evaluation metrics between training and validation data [61]. Node dropout refers to the random dropping of nodes and their connections to other layers in a model to produce a “thinned” network and prevent overfitting [62]. Strategies for combatting imbalanced training data often involve either the random repetition of minority class samples, known as oversampling, or the random elimination of samples in the majority classes, known as undersampling [63]. Oversampling is beneficial in small datasets as it retains all unique samples but can increase the chance of overfitting, whereas undersampling reduces the dataset size and, thus, the number of available unique samples [63].

The goal of this study was to develop effective NN models that can predict patient PML status and identify the tissue source from which the virus was isolated using the already established NCCR dataset created by Wilczek et al. [32]. Given the selected feature encoding methods of this research (*k*-mer frequency, text frequency-inverse document frequency, and Word2Vec) do not preserve spatial data, the MLP architecture was chosen as the basis for predictive networks. Several techniques were used to create MLP models that reduced overfitting and improved generalizability to accurately predict missing sequence data from the dataset. These techniques included using repeated *k*-fold cross-validation to determine the optimum *k*-mer size and vectorization methods for encoding NCCR sequence information, node dropout, and early stopping. The most effective model was then used to predict missing PML status and tissue isolation information in the NCCR dataset. The dimensionality of the 6-mer frequency matrix was then reduced using singular value decomposition (SVD) to visually understand the relationships between NCCR mutations and clinical manifestations. Ultimately, the MLP models yielded accurate predictions for the disease status and tissue source associated with NCCR sequences, which aided in determining how JCPyV NCCR sequences vary by patient PML status and viral tissue source. This data will provide additional context to understand how the role of machine learning can be utilized in clinical analysis and how the hypervariable region of the JCPyV genome is implicated in viral pathogenesis and tissue tropism throughout infection.

## 2. Materials and Methods

### 2.1. Query and Acquisition of JCPyV NCCR Sequences

JCPyV NCCR sequences with associated GenBank accession numbers, patient PML status, and viral tissue source were previously acquired from NCBI as described by Wilczek et al. [32]. Briefly, a custom Perl script retrieved sequences and isolated the NCCR from each GenBank accession number based on this nucleotide sequence [32].

### 2.2. Vocabulary of k-mers

NCCR sequences were transformed into various *k*-mers or substrings of the genetic sequence. An NCCR sequence of *B* base pairs and a *k*-mer length of *k* using a stride length of one nucleotide will generate a collection of L=B−k+1 *k*-mers with a theoretical 4*^k^* unique *k*-mers, as there are four unique nucleotide monomers. Each unique *k*-mer can be indexed by natural numbers in the set $K=\{1,\;2,\;3,\ldots ,\;4^{k}\}$. Because the number of nucleotides *B* varies with each NCCR sequence, so does the size of each *k*-mer collection *L*. An NN relies on numerical input of uniform length, which requires the input $x_{i}\in K^{L}$, or the collection of *L k*-mers in the *i*-th NCCR, to be mapped into a vector of features $h_{i}\in R^{W}$ of uniform length *W* features through feature extraction before NN processing. The impact of *k*-mer sizes 4-, 6-, and 8-mer on NN performance was measured to determine the *k*-mer size that maximized model accuracy while minimizing computational costs.

### 2.3. Bag-of-Words Normalized Frequency

The bag-of-words (BoW) model was used to construct a *k*-mer count matrix based on the corpus of nucleotide sequences decomposed into *k*-mer collections. First, the corpus of N *k*-mer collections $x={\left[x_{1},\;x_{2},\ldots ,\;x_{N}\right]}^{T}$, where $x_{i}$ denotes the collection of *k*-mers in the *i*-th NCCR, is used to construct an unordered set of vocabulary of length *W* representing all available *k*-mers in the corpus. The model then generates a count matrix C where $C\in N_{0}^{N\times W}$, in which the (*i*,*j*)-th entry, indicated as $c_{i,j}$, denotes the number of occurrences of the *j*-th *k*-mer in the *i*-th NCCR sequence. The BoW feature encoding was performed using CountVectorizer from the Python scikit-learn library v1.3.2 [64]. To prevent bias based on the length of the NCCR sequence or the total number of *k*-mers included in each sequence, *k*-mer frequencies were normalized across each NCCR. Let ⊘ denote elementwise division and $J_{W}$ the all-ones matrix of dimensions W×W, then:

(1) $$\hat{C}=C⊘CJ_{W},$$

where $\hat{C}$ denotes the row-normalized BoW matrix, meaning the sum of each row is one and each value ${\hat{c}}_{i,j}$ can be alternatively represented as:

(2) $${\hat{c}}_{i,j}=\frac{c_{i,j}}{\sum_{j=1}^{W}c_{i,j}}.\;$$

### 2.4. Text Frequency-Inverse Document Frequency

Like the BoW model, the text frequency-inverse document frequency (TF-IDF) is a feature extraction method that takes a corpus of texts and generates a term importance matrix $D\in R^{N\times W}$ using the term count, or term frequency, matrix C. The inverse document frequency vector $f=\left[f_{1},\;f_{2},\;\ldots ,\;f_{W}\right]$, where the *j*-th element $f_{j}\in R$ reports the uniqueness of *k*-mer *j* across the corpus of N NCCR sequences, is defined as

(3) $$f_{j}=\log\left(\frac{N+1}{N_{j}+1}\right)+1,$$

where $N_{j}$ is the number of NCCR sequences that contain the *j*-th *k*-mer. The TF-IDF term for each *k*-mer is then calculated. Let ⊙ denote element-wise multiplication and $J_{W,1}$ the all-ones matrix of dimensions W×1, then:

(4) $$D=C⊙\left(J_{W,\;1}f\right).$$

TF-IDF calculations were performed using the TfidfVectorizer from the Python scikit-learn library v1.3.2 [64]. As with the BoW model, the rows of D must be normalized such that the elements sum to one, which can be achieved by:

(5) $$\hat{D}=D⊘DJ_{W},$$

where $\hat{D}$ is the row-normalized TF-IDF matrix.

### 2.5. Word2Vec

The Google-developed NLP feature extraction tool Word2Vec embeds each word into dense vector space representations from a provided corpus based upon semantic similarities using stochastic gradient descent and backpropagation [65]. For embedding, the following parameters in the Word2Vec model were used for analysis: a word minimum count of one (min_count = 1), a vector size of 300 (vector_size = 300), a window size of 24 (window = 24), and a skip-gram model enabled (sg = 1). The parameters were previously determined by Ren et al. [55] and confirmed through exploratory experiments to optimize the efficacy of the Word2Vec model using *k*-mer information. The Word2Vec model was employed in Python using gensim v4.3.2 [66].

### 2.6. Dataset Preparation

Before model training, the dataset of NCCR sequences (*n* = 989) was subset to remove sequences with missing or irrelevant observations pertaining to the model. For models trained on classifying PML status, 348 sequences had imprecise or no PML status labels (i.e., “Suspect of PML” or missing disease status) and were excluded from model training. A total of 641 sequences were included for PML prediction models, of which 437 were labeled “PML”, 179 were labeled “Healthy”, and 25 were labeled “No PML”. The “No PML” samples are NCCR sequences from the urine of patients who received kidney transplants. To increase the accuracy and validity of the training sample, the “Healthy” and “No PML” were pooled into a single “No PML” label of 204 samples.

For models trained for predicting tissue sources, a total of 926 sequences were included for evaluation. Within the dataset, there were 566 samples labeled “Urine”, 217 labeled “CSF”, 42 labeled “PBMC”, 39 labeled “Plasma”, 32 labeled “Brain”, and 30 labeled “serum”. Samples labeled “CSF” and “Brain” were pooled into a single “CSF” group containing 249 samples. Likewise, sequences labeled “PBMC”, “Plasma”, and “serum” were pooled into a single “Blood” class containing 111 samples.

### 2.7. Multilayer Perceptron Network Architecture

Preliminary studies into JCPyV NCCR sequence classification indicated an MLP model performs slightly better in predictive performance than a CNN given the same training data and similar parameters. The MLP networks were constructed using the input, dense, and dropout layers from the Keras framework v2.12.0 [67] and the TensorFlow backend [68]. Each network possessed an input layer of nodes equal to the number of features in the input data. The input layer was followed by two hidden dense layers, each consisting of 32 fully connected nodes with rectified linear unit (ReLU) activation and a 25% node dropout rate. The output dense layer with softmax activation consisted of two nodes (PML and No PML) to predict PML status or three for tissue sources (Urine, Blood, and CSF). Models were trained for a maximum of 50 epochs but would stop after five epochs of unimproved validation data loss and will restore to the best weights to prevent overfitting. The loss was calculated using categorical cross-entropy for PML status and tissue source prediction. The chosen optimization function was Adam, using default values established by Kingma and Ba [69] and implemented in Keras [67].

### 2.8. Evaluation of Encoding Method Using Repeated k-Fold Cross-Validation

The predictive accuracy of MLP models trained on varying *k*-mer sizes and encoding methods was assessed using repeated *k*-fold cross-validation (CV). The process of *k*-fold CV consists of splitting the dataset into *k* equally sized subsets (or folds) for *k* rounds of training, where one fold is used for model validation and the remaining *k*-1 folds are used to train the model [58,70]. A fold number of five repeated ten times was used for analysis. Minority classes in the training data of each fold were oversampled to balance the dataset. Each fold was used to train a new MLP built using the described architecture above. Model training was evaluated based on accuracy. Accuracy (*Acc*) can be described using the number of true positive (*TP*), true negative (*TN*), false positive (*FP*), and false negative (*FN*) predictions as:

(6) $$Acc=\frac{TP+TN}{TP+TN+FP+FN}.$$

This process was repeated for ten iterations for all combinations of *k*-mer size and encoding method, resulting in nine unique combinations of *k*-mer size and encoding method for comparison for each model predicting PML status and tissue source. Pairwise Wilcoxon rank sign tests were performed to determine statistical significance between the accuracies, and *p*-values were adjusted using the Bonferroni method [71].

### 2.9. Predictive Model Training

For each model predicting PML status and tissue source, a randomized selection of 10% of the data was withheld from training as a test set. An additional randomized 10% was reserved as validation during training, and the remaining 80% was used as training data for the models. Minority classes in the training dataset were randomly oversampled to match that of the majority class to prevent poor training on unbalanced data [63]. Oversampling was performed using the imbalanced-learn v0.12.2, an open-source Python toolbox [72]. The most effective *k*-mer size and encoding method determined from *k*-fold CV were used to encode NCCR sequences to train the model. The maximum predicted value determined data classification labels for the test dataset. Model metrics, including accuracy, precision, recall, and *F*1 score using the testing dataset, were used. The precision (*Pr*) of a model classification can be described as:

(7) $$Pr=\frac{TP}{TP+FP},$$

the recall (*Re*) as:

(8) $$Re=\frac{TP}{TP+FN},$$

and the *F*1 score as:

(9) $$F1=\frac{2\times Pr\times Re}{Pr+Re}.$$

As each metric measures model performance in single-class prediction, metrics were averaged across classes for model-wide reporting.

### 2.10. Prediction of Missing Dataset Information

After determining the most efficacious combination of k-mer size and encoding method, the trained model was employed to predict missing information present in the original dataset. PML status predictions were provided using the most accurate model for predicting disease status for 307 samples that had no associated PML status and an additional 41 with labels not included in model training, including “GCN”, “JCPyVAN”, “JCVE”, “Suspected of PML”, and “neurological disorder which could be consistent with PML”, for a total of 348 PML status predictions. There were 49 samples with no known tissue source and an additional 14 with ambiguous sources such as “Plasma, Urine, CSF, Kidney”, “Kidney; Urine”, “CSF; Plasma”, and “Brain; Kidney”, for a total of 63 tissue source predictions. The NCCR sequences were converted into 6-mer representations and vectorized using the CountVectorizer trained on the whole dataset to ensure consistent input dimension size. The encoded data were then fed to models for prediction. Classification and confidence from NN predictions were recorded and added to the dataset under predicted information sections.

### 2.11. Singular Value Decomposition Dimensionality Reduction in Count Matrix

All 989 NCCR sequences were converted to *k*-mer collections with the most efficacious *k*-mer size of six, and a normalized frequency matrix was generated as described above, as the combination yielded the highest accuracy. The dimensionality of the resulting matrix was reduced to 30 dimensions through singular value decomposition (SVD) using the Python scikit-learn library v1.3.2 TruncatedSVD and the percent variance for each singular value were recorded [64]. The two singular values that explained the most variance were used to plot the *k*-mer information, and the PML status and tissue source information were used for data visualization. Disease status and tissue source determined by MLP prediction were labeled as ML to understand how these data points fell in relation to the known disease and tissue status. Figures were generated using the R package ggplot2 [73].

## 3. Results

### 3.1. Predictive Performance of Multilayer Perceptrons by Varying k-mer Size and Encoding Method

**Figure 1:**
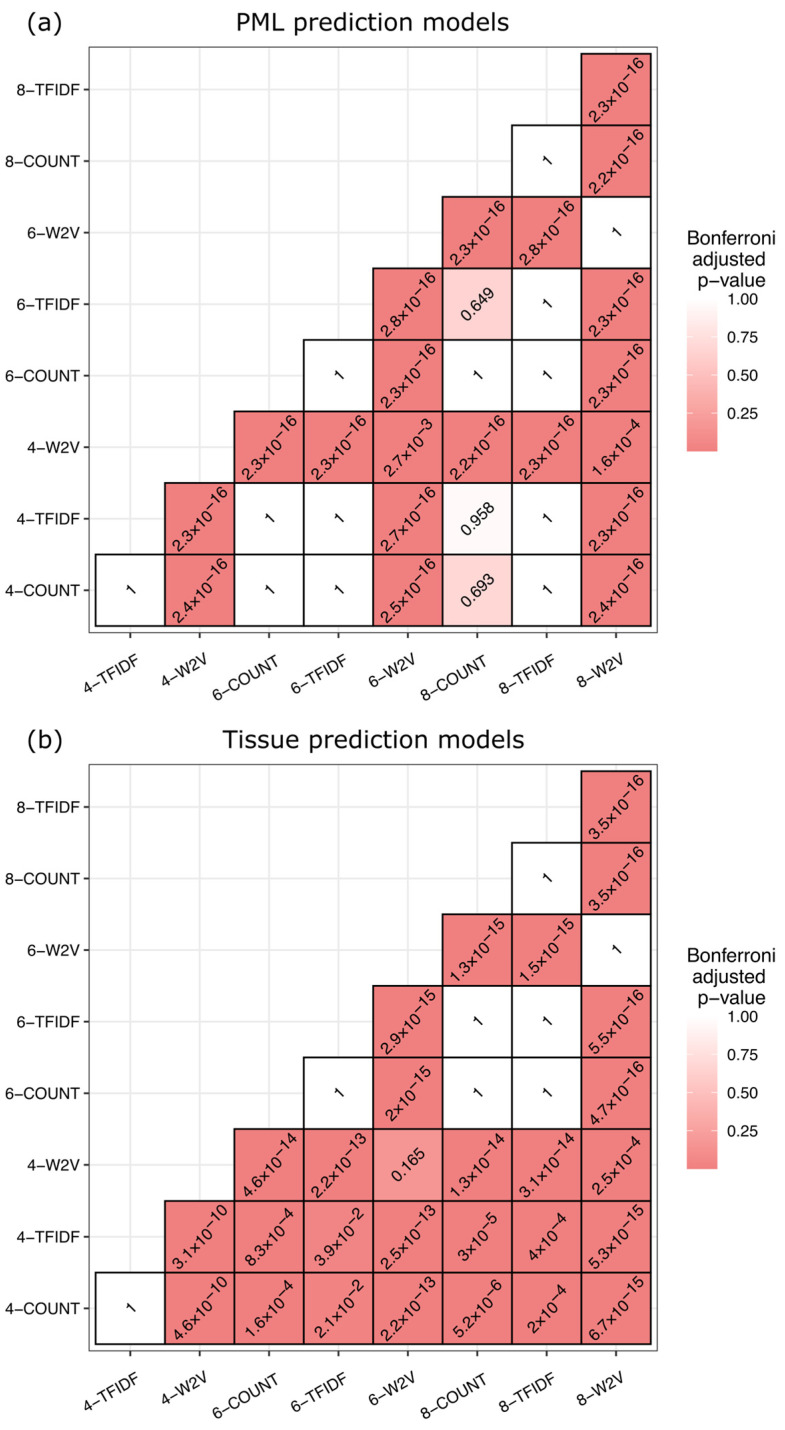
Pairwise comparison of model accuracy using varying *k*-mer size and encoding method. The heatmaps indicate the Bonferroni-adjusted *p*-values comparing the accuracy of varying *k*-mer sizes and encoding methods within multilayer perceptron model types from the pairwise Wilcoxon signed-rank test. The legend illustrates the *p*-values, where the darker red colors are *p*-values closer to zero. Accuracy was measured from ten times repeated five-fold cross-validation for each *k*-mer size and encoding method. (**a**) Statistical comparison of each *k*-mer size and respective encoding methods for PML status prediction. (**b**) Statistical comparison of each *k*-mer size and respective encoding methods for tissue source prediction. The figure was generated using the ggplot2 R package.

To determine to what extent the MLP architecture can train on the provided data, the model accuracy of PML status and tissue source prediction models trained on each permutation of 4-, 6-, and 8-mer encoded using normalized *k*-mer count frequency, normalized TF-IDF, and Word2Vec were recorded (Table 1), resulting in nine total combinations for each model type. Models were trained on the data using a five-fold CV repeated ten times, resulting in 50 folds per *k*-mer and encoding method combination. The resulting model accuracies for each combination were recorded, and the statistical significance of each combination was determined (Figure 1). The results were then used to select the encoding method for models trained to predict missing PML status and tissue source in the original dataset.

In both the PML and tissue source models, the Word2Vec encoding method yielded significantly lower accuracy than the normalized count and TF-IDF methods for all *k*-mer sizes (Table 1, Figure 1). Larger *k*-mer sizes encoded using Word2Vec yielded lower PML status and tissue source model accuracies (Table 1). The 4-mer Word2Vec model predicting PML status model exhibited significantly higher accuracy than the 6- and 8- counterparts, while the 4-mer Word2Vec tissue source model was significantly more accurate than the corresponding 8-mer model (Table 1, Figure 1). Among the PML status models, there was no appreciable difference in accuracy between the normalized count and TF-IDF methods across *k*-mer sizes (Table 1, Figure 1a). Additionally, there was no significant difference between *k*-mer sizes within the normalized count and TF-IDF encoding methods (Figure 1a). In the tissue source models, the 4-mer count and TF-IDF data performed significantly worse than the 6- and 8-mer counterparts among the tissue source models (Table 1, Figure 1b). There was no significant difference in accuracy between 6- and 8-mers within normalized count and TF-IDF data, nor a difference between the normalized count and TF-IDF using 6- and 8-mers (Figure 1b). From these results and the reduced computational costs, the 6-mer normalized count data was used as the encoding method for MLP models to predict the missing PML statuses and tissue sources in the original dataset.

### 3.2. Multilayer Perceptrons Are Capable of Accurately Predicting PML Status and Tissue Source

**Figure 2:**
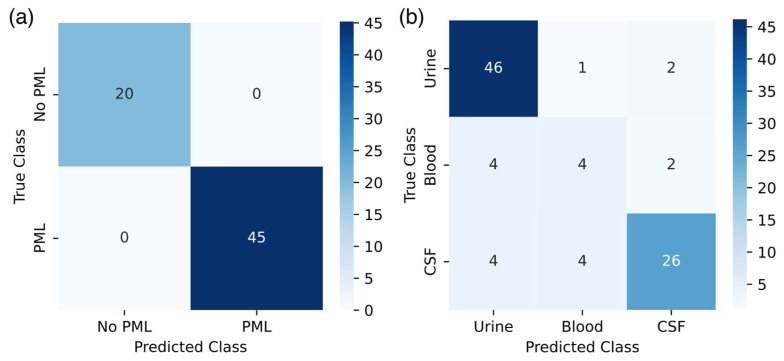
Confusion matrix of model predictions. Multilayer perceptrons (MLPs) test data classifications were recorded for model evaluation. The *y*-axis represents the real label associated with the testing data, the *x*-axis indicates the MLP-predicted labels, and the numeric value denotes the number of samples in each combination of true and MLP-predicted labels. Off-diagonal values within the same row indicate false negative predictions, while those within a column indicate false positive predictions. Darker blue values indicate greater counts of values, while lighter blue indicates fewer counts. (**a**) MLP predicts the PML status associated with a given sample. (**b**) MLP predicts the tissue source associated with a given sample. The figure was generated using the seaborn Python package.

After *k*-fold CV, new instances of each model type were established and trained on the normalized count frequency-encoded 6-mer data and evaluated using the testing data described above. The model-predicted labels based on the test dataset for each model (PML dataset, *n* = 65; tissue dataset, *n* = 93) were compared to the corresponding true labels for PML status (Figure 2a) and tissue source (Figure 2b). Results were used to determine the classic specific and macro-averaged accuracy, precision, recall, and the *F*1 score for each model (Table 2). Diagonal values denote the number of model predictions that were the same as the true labels, whereas off-diagonal values are test points incorrectly classified by the model. The PML model yielded an average accuracy of 100% and an *F*1 score of 1.00 in predicting the patient PML status for samples in the test dataset (Table 2), where the model correctly identified 45 PML sequences and 20 No PML sequences (Figure 2a). The tissue source prediction model achieved an overall accuracy of ~88% and an average *F*1 score of 0.709 (Table 2). While there was little variability in the accuracy among the different tissue sources, the *F*1 score varied greatly by class, ranging from 0.893 in the urine to 0.421 in the blood (Table 2). Of the 49 urine samples, 10 blood samples, and 34 CSF samples included in the test set, the model correctly identified 46 urine samples, four blood samples, and 26 CSF samples (Figure 2b).

### 3.3. Predicting Missing Dataset Information for Visualization

Models trained to predict PML status and tissue source were employed to predict missing information in the original dataset for further analysis. High-confidence label predictions were defined as predictions with a greater than 0.9 expectancy value. There were 348 of the original 989 samples (35.2%) that had no or imprecise PML status labels. Of the 348 sequences, 259 (74.4%) were predicted to be No PML, with 214 being high confidence, and the remaining 89 sequences (25.6%) were predicted to be PML samples, with 66 of which being high confidence (Table 3). There were 63 samples from the 989 sequences (6.4%) with either no label or an imprecise tissue source label. Of those 63 sequences, 8 were predicted as urine samples (12.7%), with 3 being high confidence, 13 as blood samples (20.6%), with no sequences being high confidence, and 42 as CSF (66.7%), with 17 being high confidence (Table 3).

**Figure 3:**
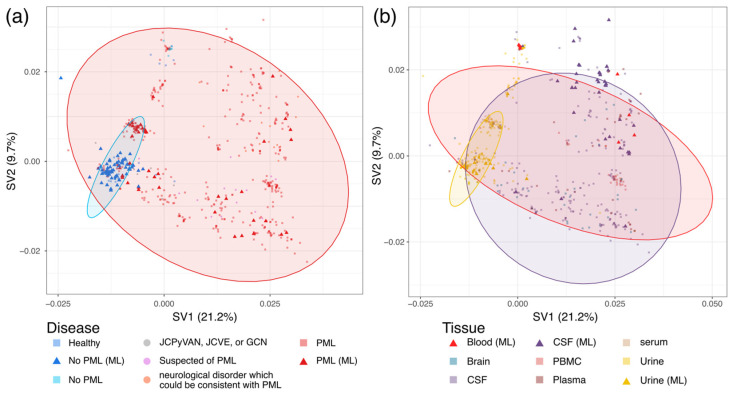
Singular value decomposition (SVD) of JC polyomavirus (JCPyV) non-coding control region (NCCR) 6-mer normalized frequency data. SVD was performed on the 6-mer normalized frequency data of the 989 JC polyomavirus non-coding control region. The two singular values explaining the greatest variance within the data were used to plot the data. Square points indicate sequences included in the training of neural network models. Circular points indicate those not included in model training, and triangular points indicate neural network-predicted labels for samples with no associated label. The sequences, including neural network training, were used to establish 95% confidence ellipses, which were overlaid on the plots. (**a**) SVD of 6-mer normalized frequency data labeled with PML status information; ellipses indicate a 95% confidence interval of training labels: No PML (blue) and PML (red). (**b**) SVD of 6-mer normalized frequency data labeled with tissue source information. Ellipses indicate a 95% confidence interval of training labels: urine (yellow), blood (red), and CSF (purple).

An SVD of the normalized 6-mer frequency was conducted to understand the biological significance and relevance of the newly predicted information. The two singular values explaining the greatest variance, 21.2% and 9.7% of the total variance, were used to plot the data (Figure 3). Points were labeled using the PML status (Figure 3a) or tissue source (Figure 3b). Labels used to train neural networks were used to construct 95% confidence ellipses and overlaid on the plot to compare how the neural network predicted labels of missing information spatially compared to true labels. The SVD depicts a greater degree of variation in the PML samples (red points) as opposed to the no PML samples (blue points), and the PML confidence ellipse almost entirely encapsulates the no PML ellipse (Figure 3a). Many predicted no PML samples (blue triangular points) fell within or near the 95% confidence ellipse, and most predicted PML samples (red triangular points) fell within the PML confidence ellipse, validating the ML predictions. When examining the SVD of the tissue data points, the urine confidence ellipse overlaps with the blood and CSF ellipses; however, the urine data points were distinct from the other two tissue types (Figure 3b). Similarly, there is a significant overlap between the blood and CSF confidence ellipses, and compared to the urine populations, these two class samples are more interspersed (Figure 3b). Overall, many of the NN-predicted PML and tissue source labels fell inside of or within proximity to their corresponding confidence ellipse, suggesting the similarity of unknown sequences to known labeled sequences and high model fidelity in predicting clinical information for NCCR sequences lacking proper labeling (Figure 3b).

## 4. Discussion

Mutations in the NCCR of the JCPyV genome have been associated with the fatal demyelinating disease, PML [30], yet few studies have explored the implications of NCCR mutations in disease due to small sample sizes [74] and nonuniformity of sample labeling. This study addresses this gap using machine learning to predict the missing patient PML status and viral tissue source in a previously compiled dataset of 989 JCPyV NCCR sequences from GenBank [32], some of which possessed insufficient sample metadata that limited the scope of inquiry. A few samples lacked the patient’s PML status or viral tissue source, while others had other information missing, such as the patient sex or geographical location. Though previous studies have demonstrated the difference in seroprevalence between sexes and the variability of genomic sequences across human populations, these variables were not included in the analyses as a result of incomplete data. Missing PML status and viral tissue source of the NCCR sequence were classified using various *k*-mer representations and encoding methods, resulting in varying degrees of accuracy depending on the selected *k*-mer size and the feature encoding method. Ultimately, the normalized 6-mer frequency was selected for feature encoding because of its high performance in predicting patient PML status and viral tissue source while reducing the computational complexity of encoding the NCCR sequences.

Mutations in the NCCR primarily consist of large duplications, deletions, or rearrangements of the block code sequences [29,30]; thus, the goal of feature embedding was to capture these events in some capacity. In generating *k*-mers for the BoW model, a stride length of one bp preserves some local ordinality between combinations of nucleotides, which may be useful in detecting unique sequences near rearrangement sites at the end of the NCCR blocks (“a” through “f”). However, the BoW model does not preserve larger or global ordinality of *k*-mer sequences and thus cannot derive inferences from the entire NCCR structure when embedding features. Though larger *k*-mer sizes would preserve greater ordinality within the NCCR sequences, there would be exponentially more possible *k*-mers, resulting in greater model complexity and computational costs. The Word2Vec method included some larger ordinality of NCCR sequences using a sliding window of *k*-mers for feature encoding, yet the process yielded significantly lower accuracy than the normalized count and TF-IDF BoW models (Table 1, Figure 1). Differences in *k*-mer size within the normalized count and TF-IDF encoding methods did not lead to any significant difference in model performance in predicting patient PML status (Figure 1a) but did have a substantial impact on predicting viral tissue sources (Figure 1b). This disparity may indicate some NCCR recombination or mutation events occur in specific tissues at a greater frequency and require greater ordinality for association, given that larger *k*-mer sizes were generally associated with improved accuracy. Tissue sources may be more difficult to associate due to the disseminated viral quasispecies undergoing frequent mutations [29].

Our results of assessing model accuracy using an MLP network in predicting patient PML status using NCCR sequences were highly accurate, sensitive, and precise (Table 2). Though the average model accuracy was 100% using the test dataset of unseen sequences, the perfect prediction score may be due to a small testing size, and the true accuracy of the model is likely to be lower than reported. The high success rate of the model can likely be attributed to the fact that almost all NCCR isolates from PML patients contain mutations and rearrangements [8]. In contrast, sequences from the urine of non-PML patients closely resemble the archetypal strain [9,12]. The prediction of samples missing PML status yielded many sequences with high confidence results (Table 3), and many of the predicted labels for NCCR sequences missing PML status information fell within or near their respective confidence ellipse (Figure 3a), providing further assurance in the classifications.

The viral tissue prediction model exhibited high fidelity in identifying urine samples (Figure 2b, Table 2), likely because of the uniformity of NCCR sequences (i.e., the lack of significant rearrangement events) isolated from urine and variability in NCCR sequences from other tissues [9,12,29]. The model exhibited difficulty correctly classifying blood samples, as more than half of the blood samples from the test dataset were falsely labeled as urine or CSF samples (Figure 2b), though it still performed better than the 33% random chance of correct classification. This difficulty in labeling the samples resulted in comparatively low precision, recall, and *F*1 scores (Table 2) and coincided with low-confidence predictions in samples missing viral tissue sources (Table 3). The blood class had an elevated accuracy compared to the other class metrics (Table 2), which likely arose from the inclusion of TN samples in the calculation (Equation (6)), where the TN includes the correctly classified urine and CSF samples. A more representative model accuracy may be derived from a test set with a balanced sampling from each class, increasing the number of blood and CSF sequences. Given the overall model performance, there is some indication that NCCR *k*-mer composition coincides with the tissue or origin. However, it is difficult to determine which features, or *k*-mers, provide the greatest contributions to model classification due to the black-box nature of NNs, making further insights into model classification decisions difficult [75].

Features of the NCCR sequences were further explored using spatial visualization of the dimensionally reduced 6-mer normalized frequency matrix via SVD, revealing distinct groupings by patient PML status and viral tissue source (Figure 3). From the SVD plot depicting PML status, there is an apparent tight grouping of No PML sequences and a resulting small 95% confidence ellipse (Figure 3a), indicating that the No PML NCCR sequences exhibit minimal *k*-mer variation. This clustering corroborates with NCCR sequences of healthy individuals undergoing infrequent mutation [9,12]. Conversely, the PML sequences were much more spread out, and this greater variation was reflected in a larger 95% confidence ellipse (Figure 3a). The greater variation in NCCR *k*-mer composition aligns with detecting mutations in PML-associated NCCR sequences [25,29,30]. Combined with the tissue classifications, there appears to be a population of sequences from the urine of PML patients within the urine 95% confidence ellipse adjacent to, yet distinct from, sequences from the urine of No PML patients (Figure 3), suggesting the emergence of quasispecies in the urine of PML patients.

The SVD plot also revealed the grouping of NCCR sequences according to the associated viral tissue source. A small grouping of urine sequences encircled within the 95% confidence ellipse (Figure 3b) coincides with the stable genetic architecture of the archetype sequence isolated from the urine of PML and non-PML patients [9,12]. There is also a significantly more extensive, yet narrow, confidence ellipse that almost completely encapsulates the urine ellipse, though there are relatively few blood and CSF samples in this area (Figure 3b). The CSF 95% confidence ellipse shares a sizeable intersection with the blood 95% confidence ellipse (Figure 3b), which appears to relate to the increased mutations associated with PML patients [29,30]. The shared variation between blood and CSF samples may be related to the model confusion in falsely classifying blood samples as CSF (Figure 2b). However, the smaller diversity and variation in the *k*-mer composition of blood sequences appear distinct from CSF samples (Figure 3b), suggesting a progression of mutational events in the NCCR from the urine to the blood to the CSF tissue types.

The results of this research were constrained by the nature of NNs, and specifically the MLP architecture, alongside the chosen methods for feature encoding. The MLP architecture was selected over other established NN architectures, primarily the CNN, as the vectorization methods chosen in this study do not preserve the ordinality of nucleotides in the original NCCR sequences. However, previous work by Câmara [42] et al. established an accurate CNN that used image representations of genomic 6-mer data to classify viral sequences, primarily SARS-CoV-2 sequences, by family, genera, and subgenus. Although the CNN achieved high accuracy, the architecture was not selected for this current study as the network relies on shared weights and local connections in the data [76], yet converting nucleotide sequences into *k*-mer representations discards the ordinality of information.

Conversely, Miao et al. [47] produced DeePhaier, an NN that was capable of using bacteriophage genomic sequences to predict phage lifestyle. The network consisted of parallel multilayer self-attention neural networks trained using one-hot encoded 300 bp segments of sequential nucleotide sequences. Although the one-hot encoding method preserves local ordinal features in sequences, one-hot encoding requires sequences to be of a fixed and consistent length. Likewise, the self-attention network architecture greatly benefits from data with ordinality and relevant sequential information. Given that the JCPyV NCCR undergoes frequent rearrangement in PML patients, resulting in variable NCCR sequence length, one-hot encoding would not serve as a viable option for sequence feature encoding.

To determine, predict, and classify SARS-CoV-2 genomes with respect to spatial and temporal changes, Sung et al. [48] created AutoCoV, a NN that leverages an auto-encoder to extract features from preprocessed genomic *k*-mer data before producing predictions on viral sequence geographic location and time period using a fully connected NN. Viral sequences were converted into *k*-mer frequency representations, considering each possible *k*-mer nucleotide combination. An entropic filter was applied to exclude *k*-mers with a low entropy value from analysis, as many *k*-mer counts will be nearly identical across genome samples. The BoW model employed in this current study only includes *k*-mers detected in a sequence within the collection, thereby automatically screening *k*-mers that are insignificant for analysis. Due to the hypervariable nature of the NCCR, differences in *k*-mer composition between sequences are inherently diverse, limiting the value of screening that has already included *k*-mers from analysis.

As several limitations remain with this approach to analysis and evaluation, future studies will be required to understand further the connections between the natural polymorphisms in the JCPyV NCCR and viral pathogenesis. NNs require a large volume of balanced training data to produce accurate predictions and provide adequate assessments, and using a small, unbalanced training dataset for predictive MLP models, such as the one employed in this study, offers several constraints. First, a relatively small dataset affects the generalizability of a model as the number of available unique cases in training is limited and may not adequately represent the diversity within the problem domain. As a result, the model may not recognize underlying patterns within the underrepresented classes, which can impact the sensitivity of the model. Second, using a small dataset increases the risk of the model overfitting to the provided training samples rather than developing generalizations, limiting the applicability of the model to provide predictions on novel samples. Third, a small dataset can limit model size and complexity as a measure to prevent overfitting, which may ultimately diminish the analytical power of the model. PML remains a rare disease, and JCPyV samples are not always sequenced or published online, further limiting the data available to train models.

Though the MLP models included in this study could achieve accurate predictions, further refinement of the models would require a larger body of well-documented, consistently formatted NCCR sequences with parity in class representation for model training to improve the predictive power. Model evaluation would also benefit from a larger testing dataset with greater representation, as small class sample sizes, such as the blood class in the tissue model test dataset, may not be entirely representative of model performance. In this study, model metrics were reported using macro-averages where class-specific metrics were weighed equally when averaging to prevent the number of class samples from biasing the resulting model averages. Additionally, online viral sequence entries capture only a portion of the metadata and can lack critical data for further analysis, such as the sex of the patient or the geographic location associated with the sample, which could be leveraged to improve the understanding of viral pathogenesis in a broader context.

Generally, NNs lack transparency in the decisions behind classifying data due to their black-box nature, making deeper analysis of the model complex. To unravel model decisions in PML and tissue classification, feature importance can indicate the relative importance of *k*-mers, allowing for the interpretation of which specific subsequences correlate with PML status and tissue source prediction. Additionally, this research described predictions using only an MLP network trained on sequence *k*-mer data, but other emergent network architectures, such as the Kolmogorov-Arnold Network [77] or the forward-forward algorithm [78], may provide the framework for networks with improved accuracy and more innate interpretability. Lastly, alternative natural language processing encoding methods, including autoencoders such as BERT [79], can be considered to preserve both global and local patterns within the NCCR sequence data.

## 5. Conclusions

JCPyV establishes a persistent, asymptomatic infection of the kidneys in most of the population and, in cases of immunosuppression such as those with HIV/AIDS or those taking immunomodulatory therapies, can cause the fatal disease PML for which there is no approved treatment. The NCCR within the JCPyV genome is a hypervariable region where rearrangements are associated with disease prognosis. Though connections between mutations in the NCCR of the JCPyV genome and PML pathogenesis have previously been established, relatively few extensive studies address the effect of NCCR mutations on PML pathogenesis and tissue tropism. This study aimed to establish MLP models capable of reliably classifying JCPyV NCCR sequences as being derived from PML or non-PML patients, identifying the tissue from which the virus was isolated, and visualizing sequence similarity through dimensional reduction techniques using a previously compiled and curated dataset of 989 JCPyV NCCR sequences [32]. Various *k*-mer sizes (4, 6, and 8) and encoding methods for feature extraction (count frequency, TF-IDF, and Word2Vec) were evaluated using the accuracy of repeated *k*-fold CV to determine the most efficacious combination for PML status and tissue source prediction (Table 1, Figure 1), and the 6-mer normalized frequency was selected for performance in both PML status and tissue source prediction. Using a test dataset, the resulting PML status and tissue source MLP models achieved a final average accuracy of 100% and 87.8%, respectively (Table 2), which were then employed to predict missing information from the original dataset for further evaluation. Using SVD for dimensional reduction in the 6-mer normalized frequency matrix, NCCR sequences generally cluster depending on the PML status of the patient, and sequences from PML patients exhibit greater variability in *k*-mer composition than those from patients without PML (Figure 3a). Additionally, the NCCR sequences from the urine of patients with or without PML were less variable and more consistent in *k*-mer composition than sequences from the blood or CSF of PML patients, which displayed a greater degree of *k*-mer variability (Figure 3b). These findings further highlight the connection between NCCR mutations and viral pathogenesis. The models developed in this research outline the functionality of NNs to produce clinically relevant information and improve the understanding of viral infections from mutated viral sequences where whole sequences may not be available. Such a process would invariably benefit understanding readily transmissible and mutable viruses with large repositories of collected sequences, such as respiratory viruses, including SARS-CoV-2 and influenza virus. Ultimately, this research explores the connection between the hypervariable nature of the JCPyV NCCR and the clinical manifestations of infection through the lens of machine learning techniques.

## Figures and Tables

**Table 1:** Accuracy of disease and tissue prediction models using varying *k*-mer sizes and encoding methods determined by repeated five-fold cross-validation repeated ten times.

*k*-mer Size	Encoding Method	% Accuracy (std)	
		Disease Model	Tissue Model
4-mer	Count frequency	97.36 (1.49)	84.87 (1.90)
	Text frequency-inverse document frequency	97.41 (1.48)	84.96 (2.22)
	Word2Vec	82.79 (6.12)	75.15 (14.10)
6-mer	Count frequency	97.94 (1.32)	86.87 (1.97)
	Text frequency-inverse document frequency	97.36 (1.59)	86.29 (1.83)
	Word2Vec	77.66 (6.95)	63.39 (23.10)
8-mer	Count frequency	98.06 (1.25)	87.06 (1.74)
	Text frequency-inverse document frequency	97.50 (1.64)	86.76 (1.76)
	Word2Vec	75.85 (8.84)	56.27 (24.49)

**Table 2:** Class-specific and macro-averaged multilayer perceptron evaluation metrics.

Model Type	Class	Accuracy	Precision	Recall	*F*1 Score
PML status	No PML	1.000	1.000	1.000	1.000
	PML	1.000	1.000	1.000	1.000
	Model average	1.000	1.000	1.000	1.000
Tissue source	Urine	0.882	0.852	0.939	0.893
	Blood	0.882	0.444	0.400	0.421
	CSF	0.871	0.867	0.765	0.812
	Model average	0.878	0.631	0.701	0.709

**Table 3:** Predictions of PML status and tissue source for missing information.

Missing Labels	Predictions (*n*, %)	High Confidence (%)
PML status (*n* = 348)	No PML (*n* = 259, 74.4%)	214 (82.6%)
	PML (*n* = 89, 25.6%)	66 (74.2%)
Tissue source (*n* = 63)	Urine (*n* = 8, 12.7%)	7 (87.5%)
	Blood (*n* = 13, 20.6%)	0 (0%)
	CSF (*n* = 42, 66.7%)	17 (40.5%)

## Data Availability

JC polyomavirus non-coding control regions were retrieved from NCBI. Materials used in the analysis are available online at https://www.mdpi.com/article/10.3390/ijms23105699/s1 (accessed on 1 May 2023).
